# The Efficacy of Kohl (Surma) and Erythromycin in Treatment of Blepharitis: An Open-Label Clinical Trial

**DOI:** 10.1155/2022/6235857

**Published:** 2022-01-24

**Authors:** Esmat Karbassi, Ehsan Amiri-Ardekani, Alireza Farsinezhad, Armita Shahesmaeili, Yasaman Abhari, Mahsa Ziaesistani, Noushin Pouryazdanpanah, Ali Derakhshani, Fatemeh Jamshidi, Haleh Tajadini

**Affiliations:** ^1^Neuroscience Research Center, Institute of Neuropharmacology, Kerman University of Medical Sciences, Kerman, Iran; ^2^Department of Phytopharmaceuticals (Traditional Pharmacy), Faculty of Pharmacy, Shiraz University of Medical Sciences, Shiraz, Iran; ^3^Student Research Committee, Shiraz University of Medical Sciences, Shiraz, Iran; ^4^Research Center for Traditional Medicine and History of Medicine, Shiraz University of Medical Sciences, Shiraz, Iran; ^5^Cell Therapy and Regenerative Medicine Comprehensive Center, Kerman University of Medical Sciences, Kerman, Iran; ^6^Department of Hematology and Laboratory Sciences, Faculty of Allied Medical Sciences, Kerman University of Medical Sciences, Kerman, Iran; ^7^HIV/STI Surveillance Research Center, and WHO Collaborating Center for HIV Surveillance, Institute for Futures Studies in Health, Kerman University of Medical Sciences, Kerman, Iran; ^8^Student Research Committee, Mazandaran University of Medical Sciences, Sari, Iran; ^9^Student Association of Indigenous Knowledge, Shiraz University of Medical Sciences, Shiraz, Iran; ^10^Medical Mycology and Bacteriology Research Center, Kerman University of Medical Sciences, Kerman, Iran; ^11^Department of Traditional Medicine, School of Persian Medicine, Kerman University of Medical Sciences, Kerman, Iran

## Abstract

**Introduction:**

Blepharitis is a common and chronic form of eyelid inflammation. Blepharitis treatment aims to decrease symptoms through antibacterial effects. One of the most common treatments of eyelid diseases in traditional medicine is using kohl. This clinical trial aimed to investigate its efficacy as a complementary treatment in staphylococcal blepharitis through an open-label clinical trial.

**Materials and Methods:**

Thirty patients were randomized to receive kohl in one eye contralateral and erythromycin ointment in another eye for 90 days. At baseline and after 90 days of treatment, symptoms, clinical signs, and side effects of treatments were recorded. Statistical analysis was carried out using SPSS software, version 19.

**Results:**

Despite randomization, there was a significant difference between the intervention and control eyes in the baseline mean clinical score (intervention eye: 9.86 (2.95) and control eye: 4.30 (2.81), *P* < 0.001). The degree of reduction of related signs and symptoms in the eyes treated with kohl was significantly higher than that in the control group: (5.2 vs. 2.20, *P* < 0.001) for symptoms and (7.40 vs. 2.46, *P* < 0.001) for clinical signs. Cohen's *d* statistic for mean difference of sign and symptom was 2.4 and 1.75, respectively, indicating a very strong effect.

**Conclusion:**

The present study results demonstrated a significant improvement in blepharitis-related signs and symptoms. The degree of improvement in the eyes treated with kohl was much higher than that in the control eyes.

## 1. Introduction

Blepharitis is one of the most common eye diseases that an ophthalmologist faces in the clinic. The etiology of blepharitis is not completely determined yet, but bacteria overload and the inflammation due to a load of bacteria have a significant role in developing the disease. Blepharitis is divided into two anterior and posterior blepharitis classifications. In anterior blepharitis, anterior lid margins are involved, while posterior blepharitis happens due to meibomian glands dysfunctions [1–3].

Staphylococcal blepharitis refers to accumulation of *Staphylococcus* epidermis in the eyelid. *Staphylococcus* produces enzymes and toxins (lipase, cholesterol esterase, and different lipid acids) that are harmful for eye tissues and causes eyelid inflammation and keratitis. Built toxins cause injury to the epithelium of the adjacent areas of eyelashes and consequently lead to fibrin formation and debride segments around eyelashes. The microbe might involve deeper cells of sebaceous glands and cause pigmentation, misdirection, and loss of eyelashes [4, 5]. The most important symptoms in all types of blepharitis are burning and irritation that is more severe in the morning and relieved during the day. Other symptoms include eye itching and discomfort after working with a computer, heaviness sensation in eyelids, secretions on eyelids, dry sensation, crusted eyelids on awakening, and red eyes. Furthermore, patients might complain of contact lenses intolerance. Clinical findings, based on the type of blepharitis, include collarets around eyelashes, lids' margin thickening and hyperemia, eyelash loss and misdirection and depigmentation, telangiectasia, lid margin irregularities and hypertrophy, and pouting and plugging of meibomian gland orifices that can be resulted in complications such as marginal ulceration of cornea, conjunctival or corneal phlyctenulosis. The most crucial symptom of staphylococcal blepharitis is eye burning and irritation in the morning due to the accumulation of microbial toxins on the cornea during sleep. This symptom is gradually improved during the day with blinking and tears flow [5–7].

Treatment goals in blepharitis are decreasing symptoms, the number of bacteria in eyelid margins and inflammation as well as improving the function of meibomian gland that are achieved through washing and warm compress of eyelids, and the use of anti-inflammatory and antibacterial agents such as erythromycin, tobramycin, cyclosporine, local dexamethasone, and oral doxycycline [8–11].

Persian medicine scientists like Razus and Avicenna have mentioned therapeutic strategies for several diseases, including eye diseases, by compiling their own scientific findings and medical findings of other nations. Identification, diagnosis, and treatment of diseases have been developed for centuries. Fortunately, a comprehensive explanation of signs and symptoms of diseases has been provided in ancient Persian medicine texts. It should be mentioned that Persian medicine, due to its philosophical and logical bases, has special attention to the causes and symptoms of diseases and the process through which symptoms are improved by removing the causes of infection [12, 13].

In Persian medicine, blepharitis has been explained in eyelid diseases section. One of the most common treatments of eyelid diseases in Persian medicine texts is using kohl. Kohl, called surma in Persian, is a mineral substance with cold and dry nature. According to Persian medicine, it has several benefits. Traditional healers have paid special attention to the treatment of eye diseases, for example, applying kohl on the eyelid can prevent cataracts and eye ulcers. According to Persian medicine, kohl is an eye tonic and preserves eye health. Avicenna in Ghanoon claims that kohl destroys any type of infection and secretions resulting from intraocular lesions and maintains eye health [12, 14, 15]. Considering the mentioned points about kohl efficacy in treating ocular infections based on Ancient Persian medicine texts, we aimed to compare kohl efficacy and safety in a clinical trial with ocular erythromycin in blepharitis treatment.

## 2. Methods and Study Design

### 2.1. Subjects

This clinical trial was a prospective, three-month, self-controlled, paired eye trial that aimed to evaluate the efficiency of consuming kohl in treating blepharitis. This clinical trial complies with the CONSORT guideline.

**Figure 1:**
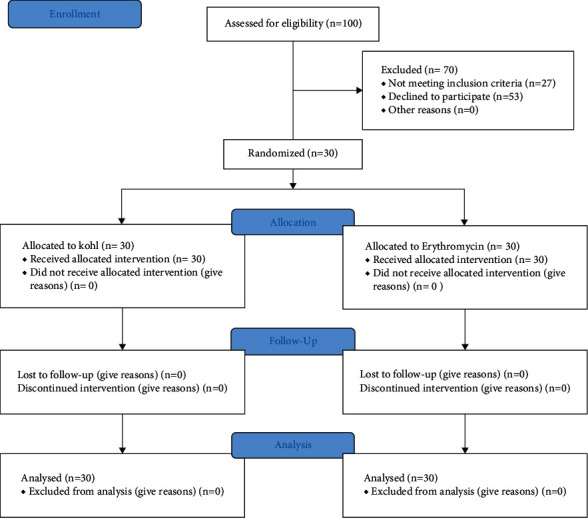
Study's consort diagram.

Participants were chosen from patients who had been referred to the ophthalmology clinic in Kerman, Iran, and diagnosed with blepharitis. There was no age or gender limitation. All patients with staphylococcal blepharitis and/or meibomian gland dysfunction were included. Patients with anterior surface problem, glaucoma, and those who did not cooperate were excluded. Pregnant cases and patients with underlying diseases were excluded. As we did not find any previous investigation on kohl efficiency on blepharitis, this study was conducted on 30 patients automatically/pilot; therefore, sample size was not measured. In this study, Random Allocation software was used to generate the eyes, left and right, to therapeutic groups, in the matter of fact the compared groups are the two eyes of the patients. Random allocation sequence was conducted by the consultant of project using the mentioned software and was given to the clinic secretary. After referring of each patient, the secretary, who had no contribution in the treatment process, would check the list and inform the physician about what treatment should be given to which eye (Figure 1).

### 2.2. Intervention

Subjects were assigned to receive kohl and erythromycin ointment in contralateral of each eye for consecutive 90 days after obtaining informed consent. All participants were treated with Fluticort^®^ (fluorometholone) eye drop twice a day and irrigation of eyelids with EyeSol^®^ shampoo every night. Kohl was provided from the local market of Mecca, Saudi Arabia, and microbial and liver toxicity tests were performed before using in this study. After confirming its safety, it was powdered and put in kohl-dan (a small pot for holding surma) to be used by cases.

### 2.3. Clinical Measurement

Clinical measurements were conducted following the TFOS DEWS II Diagnostic Methodology Subcommittee [16] at baseline, day 90 of the treatment period. Symptoms, clinical signs, and side effects of treatment were recorded in each session. The symptoms that have been assessed through self-reporting of patients were itching, burning, redness, and sensation. The clinical signs, including redness, plugin, swelling, chalazion, crust, madarosis, and inflammation, were evaluated by an ophthalmologist. All of these items scored on a 0–4 scale (Table 1). The total score for related symptoms and signs were calculated by summing up scores assigned to each item.

The treatment side effects, including increased inflammation and keratitis, were also assessed. Participants were asked to contact the clinic in case of any side effects.

### 2.4. Ethics

All patients were asked to sign the written informed consent before participation in study. For patients under 18 years, consent was obtained from their parents. The Ethics Committee of Kerman University of Medical Sciences approved this study protocol (IRCT201208017219N4, https://www.irct.ir), and the study was performed following the last version of Helsinki ethical guidelines.

### 2.5. Statistical Analysis

Statistical analysis was done using SPSS Ver.19. Mean and standard deviation of global sign and symptom scores were calculated and compared at baseline and follow-up sessions in each treatment group and between two treatments using the paired *t*-test. To measure the clinical significance of intervention, Cohen's *d* statistic was calculated. *P* values less than 0.05 were considered significant.

## 3. Result

Patients' age was from 6 to 70 years with a mean ± SD of 26.33 ± 1.64 years. Most of the participants were female (23 female and 7 male). At baseline, all eyes in the treatment of kohl and erythromycin indicated clinical signs of anterior blepharitis and meibomian gland dysfunction. The response rate at the follow-up session was 100%. Despite the random allocation of treatments, the mean score of symptoms at baseline for the intervention eye was 6.56 ± 2.45, significantly higher than that for the control eye 3.23 ± 1.73 (*P* < 0.001). There was also a significant difference between the intervention and control eyes in the baseline mean clinical score intervention eye 9.86 ± 2.95 and control eye 4.30 ± 2.81 (*P* < 0.001). After 90 days of treatment, the patient's signs and symptoms in both treatment groups significantly improved compared to baseline. Both symptom and sign scores at follow-up sessions among the eyes treated with erythromycin were lower than those in the eyes treated with kohl. Since the baseline signs and symptoms of the treated eyes were significantly more severe than the control eyes, comparing corresponding scores between the two treatment groups at follow-up sessions might be misleading. Therefore, we compared the degree of improvement in each group (calculated as differences between pre and posttreatment scores) as the outcome measure. The degree of reduction of related signs and symptoms in the eyes treated with kohl was significantly higher than the control group (5.2 vs. 2.2) (*P* < 0.001) for symptoms and (7.4 vs. 2.46) (*P* < 0.001) for clinical signs (Table 2). Also, Cohen's *d* statistic for mean difference of sign and symptom was 2.4 and 1.75, respectively, which indicates a very strong effect.

## 4. Discussion

### 4.1. Clinical Finding

The present study's findings demonstrated a significant improvement in blepharitis signs and symptoms in both treatment and control eyes. Significant reductions in symptomology scores were reported in treated eyes after 90 days. The degree of improvement in the eyes treated with kohl was much higher than that in the control eyes. Many patients affected by blepharitis with symptoms of ocular surface are important to looking for more effective management and to prevent adverse outcomes. There are many conventional treatment suggested by researchers because of not definite response to them. They suggested further studies to understanding of the etiology and associated factors and management of blepharitis.

There is no definitive cure for blepharitis, and the main goal of treatment is to reduce the symptoms as much as feasible. The duration of treatment varies depending on the severity of the disease and can differ from a few weeks to several months. Nonantibiotic treatments such as tear supplement, lid hygiene, and warm compresses are at the first line of treatment and are widely prescribed, which indicate that antibiotics are not the main treating option for blepharitis [17]. Topical antibiotics such as erythromycin only affect anterior blepharitis and are not sufficient in posterior blepharitis. Furthermore, the consuming of oral antibiotics did not significantly reduce the symptoms of blepharitis [18].

According to Lindesley, review of 34 studies on chronic blepharitis has revealed that warm compress of the eyes improves patients' symptoms while they are not cured [18].

Studies on efficacy of tear supplements and eye hygiene reported reduced associated symptoms and improved eye comfort in patients [19, 20].

### 4.2. Therapeutic Effect

Badeeb et al. concluded that a culture medium containing kohl has an inhibitory effect on *Staphylococcus* growth [21]. According to Al-Kaff et al., kohl has an inhibitory effect against *Staphylococcus* [22]. Mahmood et al. reported that kohl led to increases in nitric oxide production with antimicrobial properties [23]. According to Gupta, kohl formulated has antimicrobial activity [24]. Kohl chemical composition can explain its antibacterial and anti-inflammatory properties. Chemical analysis of kohl indicates that the main elements of kohl include lead, sulphur, antimoby, carbon, iron, and zinc [25]. Based on studies, sulphur nanoparticles have bactericidal efficacy against many bacteria, including *Staphylococcus aureus* [26]; furthermore, antimoby has antibacterial activities [27]. There are zinc-dependent signaling pathways that affect reducing inflammation. Also, zinc has antioxidant activities [28, 29]. As reported, most kohl ingredients have antibacterial and anti-inflammatory effects, which explicate kohl's efficacy in improving signs and symptoms of blepharitis.

### 4.3. Safety

Many studies have been done on lead poisoning in kohl with controversial results. Most lead poisoning cases have been seen in children as a small amount of lead is found in their blood samples. Studies suggest that the poisoning has not happened in eye contact [30, 31].

Nevertheless, other studies claimed kohl is safe to use as an eyeliner and does not have toxicity [32, 33].

Due to the observed positive effects of kohl on staphylococcal blepharitis in the present study and also its beneficial effects on eyesight mentioned in Persian medicine texts, further studies regarding to kohl are suggested.

### 4.4. Limitations

Studies on the effectiveness of kohl on blepharitis have been limited, and there was no possibility of proper comparison between the results. Also, no special method was used to determine the sample size, and patients who met the inclusion criteria were included in this trial, which is one of the limitations of the study. In addition, in this study, the group treated with kohl and the group treated with erythromycin were the same, and comparison was done between the eyes of the same group of people, while if the case and control groups were selected separately, the efficacy of kohl would be better evaluated.

## 5. Conclusion

The results of this study indicate that kohl compounds and their anti-inflammatory effects can effectively reduce the symptoms of blepharitis patients. Kohl was more effective in reducing the symptoms of blepharitis than erythromycin, which makes it a good option for treating this disease. Further studies are needed to evaluate the efficacy, potential toxicity, and optimal dose and duration of kohl in blepharitis treatment.

## Figures and Tables

**Table 1:** Scoring signs and symptoms of blepharitis patients.

Symptoms^*∗*^	0	1	2	3	4
	Absent	Sometimes	Half of the times	Most of the times	Always
Redness sign	Absent	Mild	Moderate	Severe	—
Plugin sign	Clear orifices in the middle plugging <1/3 of orifices part of the lower lid	—	Plugin <1/3 to 2/3 of orifices	Plugin >2/3 of orifices	Plugin of all orifices
Swelling sign	Absent	Mild	Moderate	Sever	—
Crust sign	Clear	Cloudy	Granular	Paste like	Nonexpressible
Madarosis sign	Absent	Mild	Moderate	Sever	—
Inflammation	Absent	Mild	Moderate	Sever	—

^*∗*^Including itching, burning, redness, and sensation in the eye.

**Table 2:** Comparison of blepharitis signs and symptoms scores at baseline and follow-up sessions in patients treated by kohl and erythromycin ointment.

Scores (mean ± S.D)	Kohl-treated eyes	Erythromycin-treated eyes	*P* value
Patient reported symptoms			
Baseline	6.56 (2.45)	3.23 (1.73)	<0.001
Follow-up	1.35 (1.56)	1.03 (1.40)	0.002
*P* value^*∗*^	<0.001	<0.001	
Clinical signs			
Baseline	9.86 (2.95)	4.30 (2.81)	<0.001
Follow-up	2.46 (1.83)	1.83 (1.94)	0.007
*P* value^*∗*^	<0.001	<0.001	
Mean differences of symptoms^*∗∗*^	5.20 ± 1.97	2.20 (1.54)	<0.001
Mean differences of clinical signs^*∗∗*^	7.40 (2.40)	2.46 (1.75)	<0.001

^*∗*^Mean difference of blepharitis-related sign/symptom score at baseline and follow-up session. ^*∗∗*^*P* value of baseline and follow-up scores in each treatment groups comparison.

## Data Availability

The data used to support the findings of this study are available from the corresponding author upon request.
